# Direct large-scale synthesis of water-soluble and biocompatible upconversion nanoparticles for *in vivo* imaging†

**DOI:** 10.1039/d4ra03242j

**Published:** 2024-05-29

**Authors:** Siqi Wang, Haiyan Liang, Zihao Yang, Zhijie Wang, Biao Yang, Chichong Lu

**Affiliations:** a Department of Chemistry, School of Light Industry Science and Engineering, Beijing Technology and Business University Beijing 100048 China luchichong@btbu.edu.cn; b University of Chinese Academy of Sciences, CAS Key Laboratory for Biomedical Effects of Nanomaterial and Nanosafety, Institute of High Energy Physics, Chinese Academy of Sciences (CAS) Beijing 100049 China; c Department of Materials Science and Engineering, School of Light Industry Science and Engineering, Beijing Technology and Business University Beijing 100048 China

## Abstract

Deep tissues can be optically imaged using near-infrared windows without radiation hazard. This work proposes a straightforward one-pot method for directly synthesizing water-soluble and biocompatible upconversion nanoparticles on a large scale for *in vivo* imaging. Safety assessment, coupled with luminescence imaging in mice, demonstrates the excellent stability and promising biological applications of the upconversion nanoparticles.

## Abstract 2

Deep tissues can be optically imaged using near-infrared windows without radiation hazard.

## Graphical abstract

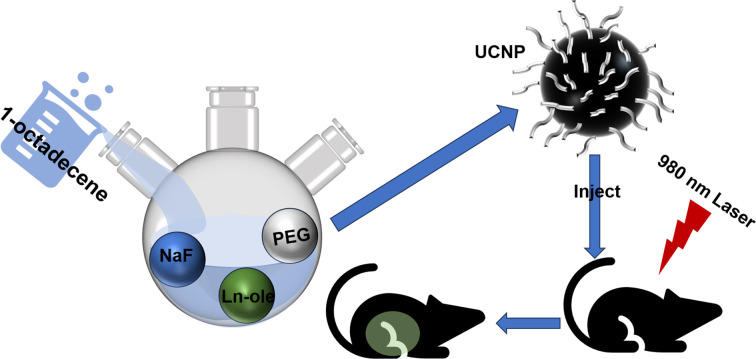

The first report of rare earth doped upconversion luminescent materials emerged in the mid-nineteenth century, wherein the incorporation of Ho^3+^, Er^3+^, Tm^3+^ and Yb^3+^ ions into a matrix led to the exciting phenomenon of infrared excitation yielding high-intensity visible luminescence.^1^ Researchers have directed their focus towards utilizing lanthanide ions as the luminescent center in upconversion luminescent materials due to their distinctive properties. However, this phenomenon was restricted to crystalline and block glass materials.

Owing to advancements in nanotechnology, the emergence of upconverted nanoparticles (UCNPs) has opened up a new avenue for research in nanoscience. These nanoparticles, typically composed of an inorganic matrix hosting rare earth ions, have garnered significant interest due to their potential applications in fields such as biomedicine, anti-counterfeiting measures, and sensing technologies.^2–6^ Rare earth-doped upconversion luminescent materials can be delineated into three structural components: activator, sensitizer, and matrix, based on their functional roles. Lanthanide rare earth ions serve as an activator, acting as the optically active center that generates upconversion luminescence.^7,8^ To enhance upconversion efficiency, activator dopants are often employed as sensitizers.^9^ The upconversion process primarily stems from the distinct energy levels of doped rare earth ions, with Nd^3+^, Ho^3+^, Er^3+^ and Tm^3+^ commonly studied as activators due to their rich electronic energy profiles and high upconversion luminescence efficiency.^10,11^ Sensitizers within the material structure primarily serve as light-absorbing antennas, absorbing near-infrared or infrared excitation light and subsequently transferring the absorbed energy to the activator.^12^ The Yb^3+^ ion emerges as a frequently utilized sensitizer owing to its substantial absorption cross-section in the near-infrared region, facilitating effective absorption of near-infrared light.^13–18^ Notably, the NaYF_4_:Ln^3+^ nanoparticles have emerged as pivotal luminescent nanomaterials for various applications, including bioimaging, optical data storage, and anti-counterfeiting measures, owing to their exceptional upconversion properties. However, the large specific surface area of NaYF_4_:Ln^3+^ nanoparticles often leads to significant non-radiative transitions, posing challenges for the exploration and application of new optical functionalities.^19,20^ By adjusting the concentration and composition of dopant ions, it becomes feasible to regulate the relative intensity of different emission wavelengths, thereby enhancing luminescence efficiency.^21,22^

The upconversion luminescence process, involving 4f electron transitions within the inner layer, does not entail the breaking of chemical bonds. Consequently, UCNPs exhibit high stability and are resistant to photofading or photochemical decay, offering notable advantages over other fluorescent materials.^23–25^ UCNPs hold immense promise for biological imaging, particularly in the near-infrared spectrum. This spectral range experiences minimal tissue attenuation, making it conducive for deep tissue imaging.^26–28^

In general, various synthesis techniques are employed to fabricate nanoparticles with controlled stoichiometry, crystal structure, and morphology.^29–33^ Among these techniques, the thermal decomposition is particularly prominent due to its ability to precisely regulate the stoichiometric content, phase, size, and morphology of UCNPs. The synthesis process is typically involving the utilization of appropriate long-chain ligands in high boiling organic solvents to ensure high crystallinity and homogeneous shape. The surface of the synthesized UCNPs is typically covered by hydrophobic ligands. However, achieving water solubility is imperative for utilizing UCNPs in biological imaging applications.

In this work, we present a straightforward method for the large-scale preparation of water-soluble and biocompatible UCNPs. The synthesized materials exhibit high stability and upconversion luminescence properties, rendering them suitable for optical imaging with excitation in the NIR window. We conducted a direct synthesis of water-soluble NaYF_4_:Yb^3+^/Er^3+^ nanoparticles, which were subsequently coated with polyethylene glycol (PEG) to enhance blood circulation time.^34^ Our approach involved a simple experimental setup capable of producing large quantities of product in a single batch without the need for complex screening and purification procedures. As shown in Fig. 1a, the synthesis process involved the straightforward mixing of 1-octatetene solvent with lanthanide oleates, sodium fluoride, and poly(ethylene glycol)bis(carboxymethyl) ether (polyethylene glycol 600 diacid). The mixture was then heated successively at 135 °C for 1 hour, followed by 200 °C for 0.5 hours, and finally at 310 °C for 1 hour under nitrogen reflux.

**Fig. 1:**
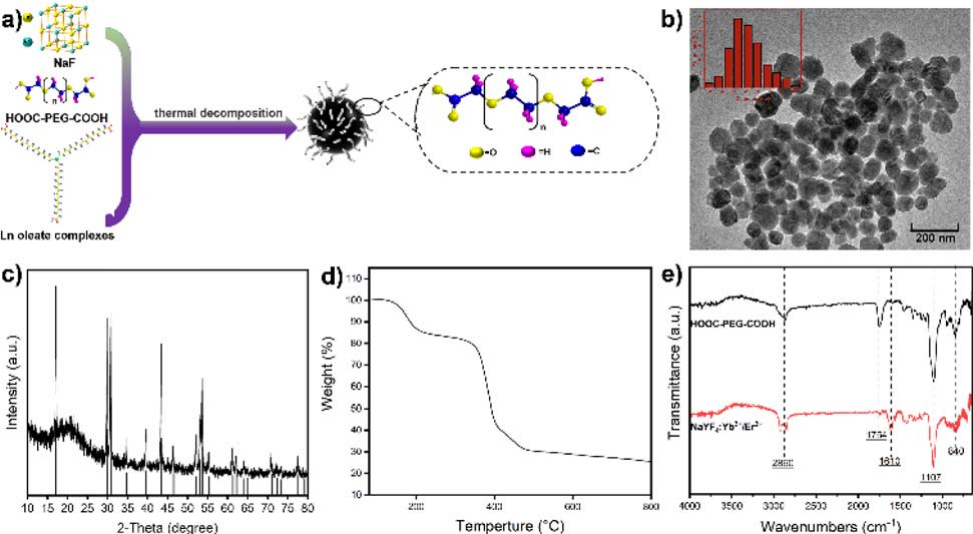
(a) Synthesis process of UCNPs. (b) TEM image of NaYF_4_:Yb^3+^/Er^3+^ (20/2 mol%). (c) XRD pattern of NaYF_4_:Yb^3+^/Er^3+^ (20/2 mol%). Bars: NaYF_4_ from the PDF card (#028-1192). (c) TGA curve of NaYF_4_:Yb^3+^/Er^3+^ (20/2 mol%). (e) FT-IR spectra of UCNPs and HOOC–PEG–COOH.

The inclusion of the flexible linear polymer PEG, with its carboxyl functional groups, facilitated covalent coupling with lanthanide ions on the nanoparticle surface. The long hydrophilic chain of the polymer prevented nanoparticle aggregation, ensuring high water solubility and colloidal stability. Furthermore, the non-toxic and anti-adhesive nature of the coating formed a hydration layer on the nanoparticle surface, preventing protein corona formation and phagocytosis, thereby significantly prolonging nanoparticle circulation time *in vivo*.

Our synthesis method offers simplicity and scalability, allowing for the production of water-soluble UCNPs ranging from milligrams up to 40 grams. Transmission electron microscopy (TEM) imaging demonstrated well-dispersed nanoparticles with an average diameter of approximately 50 nm when dispersed in water (Fig. 1b). X-ray diffraction (XRD) analysis confirmed the successful synthesis of NaYF_4_ nanoparticles (Fig. 1c), consistent with the crystal structure reported in the PDF card (#028-1192). The organic content on the surface of the sample was analyzed *via* thermogravimetric analysis (TGA), with the resulting weight loss curve presented in Fig. 1d. The data reveals that the sample exhibits an inorganic content of approximately 30%. Fourier transform infrared spectroscopy (FT-IR) analysis revealed characteristic peaks corresponding to the carboxyl group (C=O) stretching vibration at 1754 cm^−1^ and the C–O–C bond of PEG at 1107 cm^−1^.^35^ Compared with the infrared spectra of pure HOOC–PEG–COOH, a new absorption peak appears at 1610 cm^−1^, and the intensity of the absorption peak at 1754 cm^−1^ was decreased (Fig. 1e). This was probably due to the carboxylic acid in the end of HOOC–PEG–COOH binding with the nanoparticles and converting into carboxylate, and the other end is exposed to the surface in free form. This indicates that the nanoparticle surface is coated with polyethylene glycol, giving it high water solubility and biocompatibility.

The upconversion nanoparticles were synthesized with varying ratios of Yb^3+^ and Er^3+^, and the highest luminescence efficiency was observed at a molar ratio of 20 : 2 for Yb^3+^ and Er^3+^ (Fig. 2a). Upon excitation by a NIR laser, the UCNPs emitted a predominant green light. The emission spectra of the nanoparticles, stimulated at 980 nm by a continuous-wave laser, revealed primary Er^3+^ ion emissions at 526 nm, 543 nm, and 652 nm, corresponding to specific transitions: ^2^H_11/2_ → ^4^I_15/2_, ^4^S_3/2_ → ^4^I_15/2_, and ^4^F_9/2_ → ^4^I_15/2_, respectively. Increased doping of Er^3+^ ions resulted in enhanced fluorescence emission intensity. The NIR laser proved to be an efficient excitation light source for *in vivo* luminescence imaging applications due to its high tissue penetration depth.

**Fig. 2:**
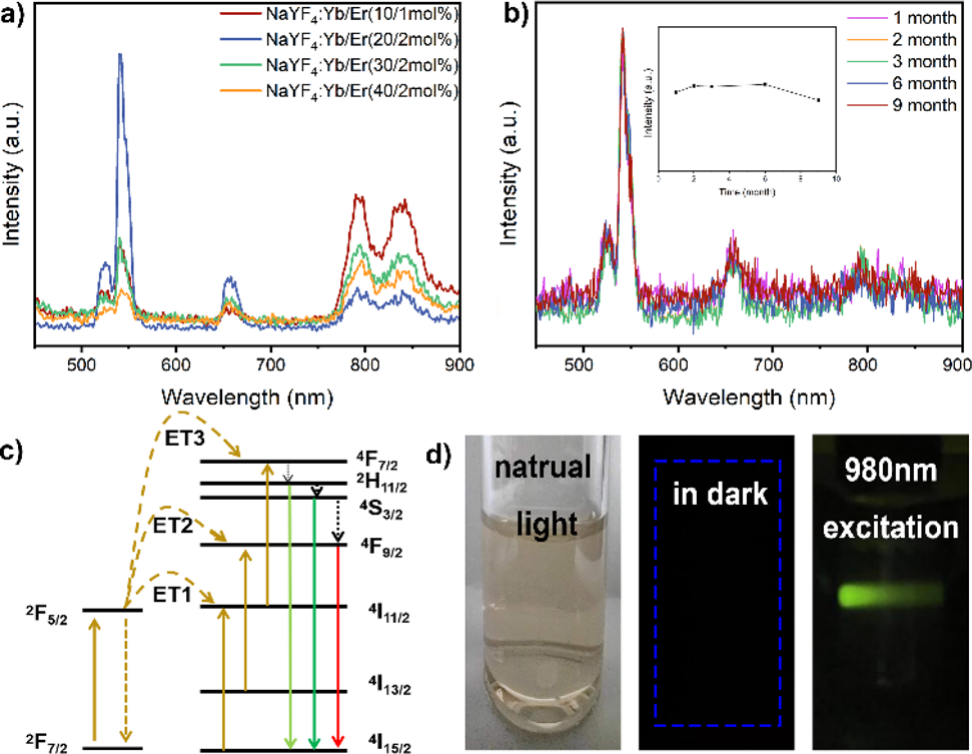
(a) Luminescence spectra of NaYF_4_:Yb^3+^/Er^3+^ nanoparticles. (b) Luminescence stability of nanoparticle colloidal solution after storage for different time. (c) Yb^3+^ to Er^3+^ energy level diagrams and related transfer processes. (d) Photographs of colloidal solutions of NaYF_4_:Yb^3+^/Er^3+^ (20/2 mol%) nanoparticles in water under different lighting conditions.

In stability experiments, the UCNPs maintained excellent emission behavior under long-term and high-intensity laser irradiation, and even after storage for 9 months, the nanoparticle colloidal solution exhibited good stability (Fig. 2b). The energy level maps of Yb^3+^ and Er^3+^, along with the upconversion process under 980 nm excitation, depicted three energy transfer (ET) processes responsible for the green (526 nm and 543 nm) and red (652 nm) emissions displayed by NaYF_4_:Yb^3+^/Er^3+^ nanoparticles (Fig. 2c). Initially, ions transitioned to the ^4^F_7/2_ state through ET1: ^2^F_5/2_(Yb^3+^) + ^4^I_15/2_(Er^3+^) → ^2^F_7/2_(Yb^3+^) + ^4^I_11/2_(Er^3+^) and ET3: ^2^F_5/2_(Yb^3+^) + ^4^I_11/2_(Er^3+^) → ^2^F_7/2_(Yb^3+^) + ^4^F_7/2_(Er^3+^).^36^ This led to non-radiative relaxation to the ^2^H_11/2_ and ^4^S_3/2_ states, followed by leaps to the ^4^I_15/2_ state, resulting in two green emissions.^37,38^ Through ET2, Er^3+^ ions were pumped to the ^4^F_9/2_ state: ^2^F_5/2_(Yb^3+^) + ^4^I_13/2_(Er^3+^) → ^2^F_7/2_(Yb^3+^) + ^4^F_9/2_(Er^3+^). Additionally, ions from the ^2^H_11/2_ and ^4^S_3/2_ states non-radiatively transitioned to the ^4^F_9/2_ state.

The chemical binding of HOOC–PEG–COOH molecules to UCNPs was facilitated by the coordination of COO-groups with Ln(iii) ions on both surfaces, ensuring strong colloidal stability and water solubility critical for biological applications. UCNPs exhibited no change in hydrodynamic size even after storage for more than 1 month in water, PBS solution simulating the physiological environment, and sodium chloride solution simulating an extremely harsh environment (Fig. 3a–c), indicating excellent colloidal stability. Moreover, the aqueous solution demonstrated remarkable colloidal stability, as evidenced by continuous monitoring of zeta potential over an extended period (Fig. 3d). Cytotoxicity assessment using the Cell Counting Kit-8 revealed high cell viability even at nanoparticle concentrations up to 500 mg L^−1^ (Fig. 3e). Blood biochemical analysis in mice injected with nanoparticles showed no significant changes compared to the control group, indicating excellent biocompatibility (Fig. 3f).

**Fig. 3:**
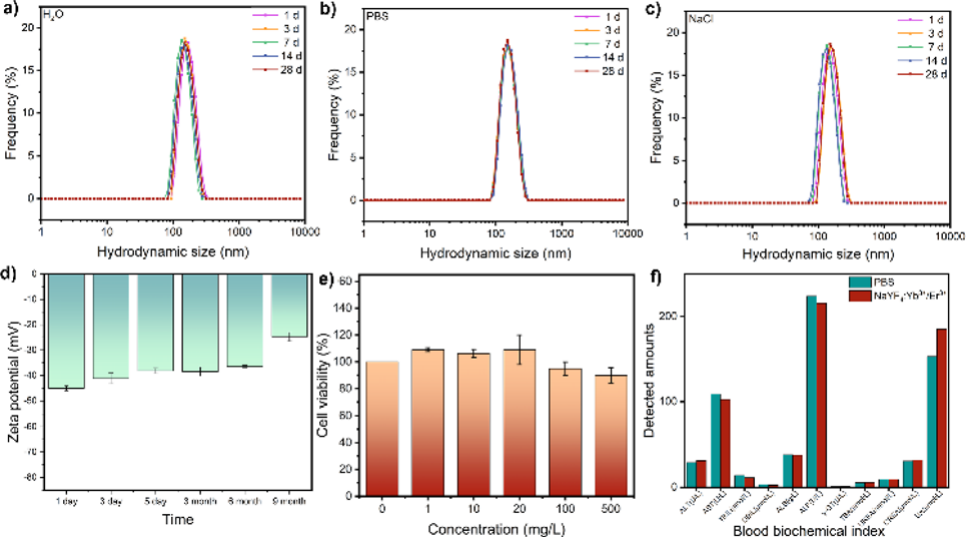
Hydrodynamic sizes of NaYF_4_:Yb^3+^/Er^3+^ (20/2 mol%) nanoparticles dispersed in various solutions: (a) deionized water, (b) PBS, and (c) NaCl solutions. (d) Long-term monitoring of zeta potential. (e) Cytotoxicity of NaYF_4_:Yb^3+^/Er^3+^ (20/2 mol%). (f) Blood biochemical changes in mice following injection of NaYF_4_:Yb^3+^/Er^3+^ (20/2 mol%) for 7 days.

After a preliminary biosafety assessment of the samples, UCNPs were administered to mice, and their activity was monitored. All animal procedures were performed in accordance with the Guidelines for Care and Use of Laboratory Animals of BTBU and approved by the Scientific Research Ethics Committee of BTBU, China. As shown in Fig. 4, no evident abnormalities were observed. There was no notable histopathological damage in the major organs of the control mice. The heart, liver, spleen, lungs, and kidneys of the experimental group exhibited general consistency with those of the control mice, showcasing normal tissue cell morphology and an absence of acute inflammatory responses. Moreover, no inflammatory reactions were detected in tissue sections taken at 24 hours and 72 hours post-injection, suggesting that neither short-term nor prolonged exposure to UCNPs induced organ damage in the mice (Fig. 4a and b). The metabolic and clearance pathways of UCNPs in mice were investigated by quantifying yttrium levels in feces and urine. The study revealed that UCNPs were primarily metabolized through fecal excretion within the initial four hours, shifting towards urinary excretion thereafter (Fig. 4c and d). Complete elimination was achieved within 72 hours.

**Fig. 4:**
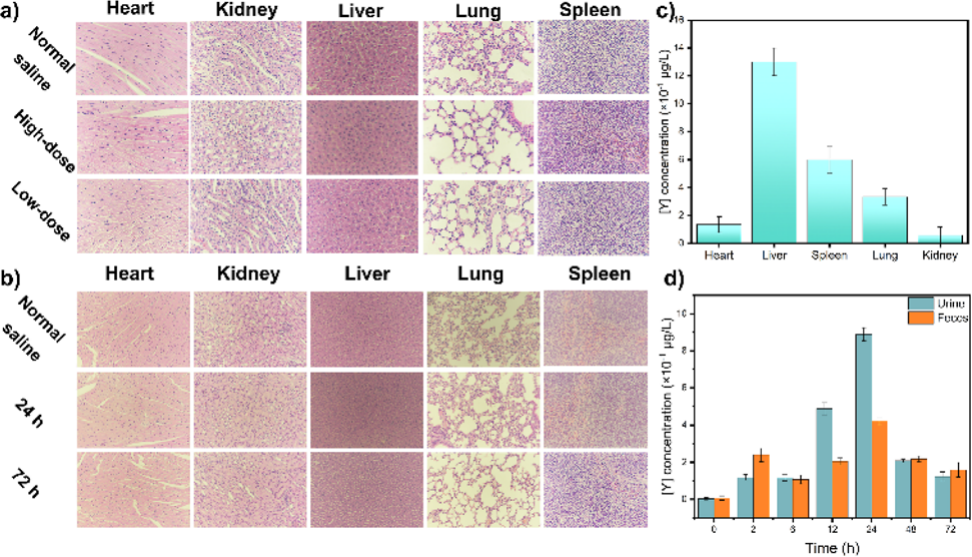
(a) Tissue sections from mice injected with different concentrations of UCNPs. (b) Tissue sections at different times after injection of UCNPs at a dosage of 2 mg [Y] per kg body weight. (c) The amount of Y in the tissue 2 hours after UCNP injection. (d) The amount of Y present in the mouse excreta.

Luminescence imaging serves as a crucial optical method, leveraging luminescent materials to capture image data of targeted entities. These materials possess the ability to emit luminescence upon exposure to excitation light, enabling the study of biological functions within living cells or tissues. The upconversion nanoparticles do not need strict phase matching, and the stability of excitation wavelength is not high, and the switching effect of upconversion luminescence can be achieved by various wavelength light regulation (Fig. S1†). *In vivo* evaluation of luminescence imaging function often involves animal models. In this study, BALB/c mice received injections of NaYF_4_:Yb^3+^/Er^3+^ (20/2 mol%) nanoparticles *via* the tail vein. As shown in Fig. 5, the nanoparticles dispersed throughout the mouse body, facilitating deep tissue imaging. Notably, the liver emerged as a prime diagnostic target due to its heightened signal. The synthesized UCNPs exhibit a myriad of advantageous properties, including water solubility, biocompatibility, high optical stability, large Stokes shift, all of which are pivotal for *in vivo* disease detection.

**Fig. 5:**
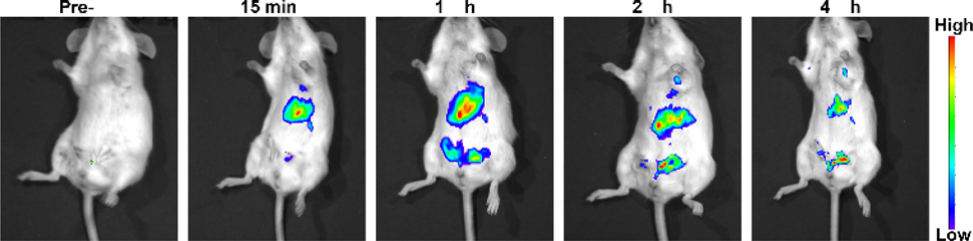
Luminescence images following injection of NaYF_4_:Yb^3+^/Er^3+^ (20/2 mol%) nanoparticles into the tail vein of a BALB/c mouse at a dosage of 1 mg [Y] per kg body weight.

In conclusion, our study effectively employed a one-pot approach to produce biocompatible and water-soluble lanthanum-doped upconversion nanoparticles on a large scale, reaching tens of grams. These NaYF_4_:Yb^3+^/Er^3+^ nanoparticles demonstrate high upconversion efficiency, excellent biocompatibility, and colloidal stability. Furthermore, their excitation wavelength in the near-infrared window enables deeper tissue penetration at lower energy levels, mitigating radiation risks associated with their regular use. The ease, speed, and simplicity of our synthesis method not only reduce manufacturing costs but also enhance productivity, rendering large-scale synthesis more feasible for industrial applications. This work lays the groundwork for the large-scale production of water-soluble and biocompatible upconversion nanoparticles, thereby fostering further exploration and rapid advancement of their structural and functional attributes in the biomedical field.

## Conflicts of interest

There are no conflicts to declare.

## Supplementary Material

RA-014-D4RA03242J-s001
