# Supplementary material for: Direct large-scale synthesis of water-soluble and biocompatible upconversion nanoparticles for in vivo imaging
Source: RSC Adv. 2024 May 29;14(25):17350–4. doi: 10.1039/d4ra03242j (PMC11134336; doi:10.1039/d4ra03242j)
Supplement: RA-014-D4RA03242J-s001 [file RA-014-D4RA03242J-s001.pdf]

Electronic Supplementary Material (ESI) for RSC Advances.
This journal is © The Royal Society of Chemistry 2024

Supplementary Information

Directly large-scale synthesis of water-soluble and biocompatible upconversion nanoparticles for in vivo imaging

Siqi Wang,[a] Haiyan Liang,[a] Zihao Yang,[a] Zhijie Wang,[b] Biao Yang,[c] and Chichong Lu[a*]

[a] Department of Chemistry, School of Light Industry Science and Engineering, Beijing Technology and Business University, Beijing 100048, China.

[b] University of Chinese Academy of Sciences, CAS Key Laboratory for Biomedical Effects of Nanomaterial and Nanosafety, Institute of High Energy Physics, Chinese Academy of Sciences (CAS), Beijing 100049, China.

[c] Department of Materials Science and Engineering, School of Light Industry Science and Engineering, Beijing Technology and Business University, Beijing 100048, China

* E-mail: luchichong@btbu.edu.cn

Experimental section

1. Reagents and Materials

The following reagents were purchased from Sigma-Aldrich: poly(ethylene glycol) bis(carboxymethyl) ether (polyethylene glycol 600 diacid, HOOC-PEG-COOH), 1-octadecene (90 %), sodium fluoride (99 %). The following reagents were purchased from Macklin: yttrium (III) nitrate (99.5 %), ytterbium (III) nitrate (99.99 %), erbium (III) nitrate (99.9 %) and n-hexane. Sodium oleate (97 %) was purchased from TCI.

2. Synthesis of Y-oleate, Yb-oleate, Er-oleate

Yttrium (III) nitrate (20 mmol, 5.5 g) and sodium oleate (60 mmol, 18.3 g) were added to a mixture of distilled water (30 mL), ethanol (40 mL) and n-hexane (70 mL). The resulting mixture was stirred at room temperature for 12 h. After completion of the reaction, the upper organic layer underwent washing three times with a mixture of

distilled water (50 mL) and ethanol (50 mL) in a separating funnel. After evaporation of hexane, the Y-oleate complex was obtained as a waxy solid. Yb-oleate and Er-oleate were synthesized using the same reaction and purification procedures as for Y-oleate.

3. Synthesized water-soluble $NaYF_4:Yb^{3+}/Er^{3+}$

Typically, $NaYF_4:Yb^{3+}/Er^{3+}$ (20/2 mol%) nanoparticles were synthesized through thermal decomposition of Ln-oleate complexes. Briefly, Y-oleate (39 mmol), Yb-oleate (10 mmol), Er-oleate (1 mmol), sodium fluoride (200 mmol) and HOOC-PEG-COOH (100 mmol) were mixed in a three-neck bottle flask containing 1-octadecene. The solution was heated to 135°C for 1 h, then at 200°C for another 0.5 h, and refluxed at 310°C for 1 h under a nitrogen atmosphere. The heating rate was maintained at 3-5°C/min during the heating process. The resulting solution was then cooled to room temperature.

4. Characterization

X-ray diffractometer (Bruker D2) operated with Cu Kα radiation (λ =1.5406 Å) at a scanning speed of 6°/min. Transmission electron microscopy (TEM) images were recorded on a JEOL JEM-2100 operating at an accelerating voltage of 100 kV. Fourier-transform infrared (FTIR) spectra were recorded using a Thermo IR200 spectrometer with a resolution of 4 cm^{-1}. The size distribution and zeta potential of the nanoparticles were measured at 25°C with a dynamic light scattering (DLS) detector (Malvern). Luminescence spectra were obtained using a fluorescence spectrophotometer (Horiba Jobin Yvon FluorolLog3) equipped with a xenon lamp and an external 980 nm laser with a power density of 85 mW/mm^2. TGA instrument (DTG-60 AH, Shimadzu) was employed under a nitrogen atmosphere.

5. Cytotoxicity

Cells in logarithmic growth phase were seeded into 96-well plates at 100 μL of cell suspension per well. 100 μL of medium containing different concentrations of the tested samples was added. After 24 h, 10 μL of CCK-8 solution was added, and culture continued for 1 h. The 96-well plate was removed, and absorbance was measured at 450 nm using a microplate reader.

Blank group: medium; control group: medium + cells + PBS; experimental group: medium + cells + tested samples.

6. In vivo toxicity evaluation

Female BALB/c mice aged 5 weeks were purchased from Beijing Vital River Laboratory Animal Technology Co., Ltd. (Beijing, China). BALB/c mice were injected with 200 μL of high-dose solution (2.0 mg [Y]/kg mouse weight) and low-dose solution (0.2 mg [Y]/kg mouse weight) through the tail vein for the experimental group, while the control group received an equivalent amount of PBS. Major organs were collected 24 and 72 hours later, sectioned and subjected to H&E staining to observe histopathological changes for tissue toxicity assessment.

7. In vivo imaging

BALB/c mice were injected with $NaYF_4:Yb^{3+}/Er^{3+}$ nanoparticles (1 mg [Y] kg-1 of mouse body weight) through the tail vein. Fluorescence images were obtained using an animal in vivo imaging system (Perkins Elmer, IVIS Spectrum, USA) with excitation at 860 nm with a power density of ~1 W/cm^2, at pre-injection, 0.25, 1, 2, and 4 h post-injection.

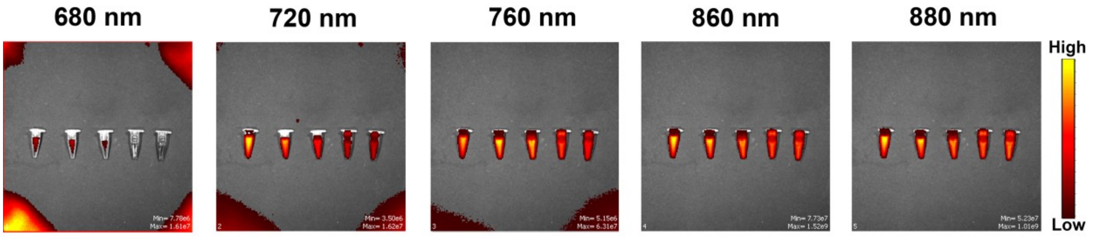

Figure S1. Luminescence intensity of sample solutions injected into mice and diluted into different concentrations (75%, 50%, 25%, and 5%) (left to right) under various excitation conditions.
